# Identification of Actin Filament-Associated Proteins in Giardia lamblia

**DOI:** 10.1128/spectrum.00558-21

**Published:** 2021-07-21

**Authors:** Melissa C. Steele-Ogus, Richard S. Johnson, Michael J. MacCoss, Alexander R. Paredez

**Affiliations:** a Department of Biology, University of Washington, Seattle, Washington, USA; b Department of Genome Sciences, University of Washington, Seattle, Washington, USA; Broad Institute

**Keywords:** *Giardia*, actin, actin-binding proteins, cytoskeleton, molecular parasitology

## Abstract

The deep-branching protozoan parasite Giardia lamblia is the causative agent of the intestinal disease giardiasis. Consistent with its proposed evolutionary position, many pathways are minimalistic or divergent, including its actin cytoskeleton. *Giardia* is the only eukaryote known to lack all canonical actin-binding proteins. Previously, our lab identified a number of noncanonical Giardia lamblia actin (*Gl*Actin) interactors; however, these proteins appeared to interact only with monomeric or globular actin (G-actin) rather than with filamentous actin (F-actin). To identify F-actin interactors, we used a chemical cross-linker to preserve native interactions followed by an anti-*Gl*Actin antibody, protein A affinity chromatography, and liquid chromatography coupled to mass spectrometry. We found 46 putative actin interactors enriched under the conditions favoring F-actin. Data are available via ProteomeXchange with identifier PXD026067. None of the proteins identified contain known actin-interacting motifs, and many lacked conserved domains. Each potential interactor was then tagged with the fluorescent protein mNeonGreen and visualized in live cells. We categorized the proteins based on their primary localization; localizations included ventral disc, marginal plate, nuclei, flagella, plasma membrane, and internal membranes. One protein from each of the six categories was colocalized with *Gl*Actin using immunofluorescence microscopy. We also co-immunoprecipitated one protein from each category and confirmed three of the six potential interactions. Most of the localization patterns are consistent with previously demonstrated *Gl*Actin functions, but the ventral disc represents a new category of actin interactor localization. These results suggest a role for *Gl*Actin in ventral disc function, which has previously been controversial.

**IMPORTANCE** Giardia lamblia is an intestinal parasite that colonizes the small intestine and causes diarrhea, which can lead to dehydration and malnutrition. *Giardia* actin (*Gl*Actin) has a conserved role in *Giardia* cells, despite being a highly divergent protein with none of the conserved regulators found in model organisms. Here, we identify and localize 46 interactors of polymerized actin. These putative interactors localize to a number of places in the cell, underlining *Gl*Actin’s importance in multiple cellular processes. Surprisingly, eight of these proteins localize to the ventral disc, *Giardia*’s host attachment organelle. Since host attachment is required for infection, proteins involved in this process are an appealing target for new drugs. While treatments for *Giardia* exist, drug resistance is becoming more common, resulting in a need for new treatments. *Giardia* and human systems are highly dissimilar, thus drugs specifically tailored to *Giardia* proteins would be less likely to have side effects.

## INTRODUCTION

Actin is a highly conserved filament-forming protein with essential roles in all eukaryotes, that include signaling, motility, membrane trafficking, cell polarity, and cytokinesis (1). Both actin monomers (globular or G-actin) and polymerized actin (filamentous or F-actin) have essential functions in the cell; therefore, the balance between the two forms has a regulatory role in addition to a structural one (2). Eukaryotes throughout the evolutionary tree possess a number of regulators which spatially and temporally control filament formation and depolymerization; their functions include monomer sequestration, filament nucleation or elongation, severing, capping, and cross-linking of filaments (1). Other actin interactors fulfill such roles as linking to organelles and the plasma membrane or moving cargo and generating contractile forces.

The protozoan parasite Giardia lamblia is the only eukaryote known to lack all of the canonical actin-binding proteins (3). Due to the lack of conserved interactors that constrain actin evolution, Giardia possesses the most divergent actin identified to date, which is only 58% identical to the average eukaryotic actin; in contrast, Saccharomyces cerevisiae actin and human skeletal actin are 87% identical (4, 5). However, Giardia actin (*Gl*Actin) retains conserved roles in many cellular processes, including membrane trafficking, cell polarity, and cytokinesis (6, 7). These conserved roles indicate the presence of noncanonical interactors in the proteome (8). The key cellular role of *Gl*Actin, as well as its extreme divergence, could make it and its interactors potential drug targets. Giardia has been designated the cause of a neglected disease by the World Health Organization, and giardiasis results in millions of cases of diarrheal disease worldwide each year (9).

Our lab previously identified a number of actin-associated proteins in Giardia (10); that study focused on proteins with conserved identifiable domains whose actin-binding function either may have been overlooked or may not exist in other organisms. Those interactors included microtubule and flagellum-related proteins such as p28 and centrin, the chaperone HSP70, the DNA helicase TIP49, the nuclear ARP7, the atypical mitogen-activated protein (MAP) kinase ERK7, and the regulatory protein 14-3-3.

Much of the Giardia genome contains genes annotated as “hypothetical,” as they do not contain any known domains and/or are unique to Giardia. Our previous study identified a number of novel actin-binding partners in Giardia (10) but did not examine these particular proteins further. This study also used a twin-strep tag to affinity purify *Gl*Actin and did not utilize buffer conditions that stabilize actin filaments; furthermore, the punctate localization of the interactors described were consistent with that of monomeric actin. Therefore, it is likely that our earlier work may have missed proteins that bind exclusively to filamentous actin and did not provide a comprehensive list of all the actin interactors in Giardia.

Here, we used a different approach to discover novel *Gl*Actin interactors for the purpose of identifying those which bind to F-actin. Using a custom anti-*Gl*Actin antibody, protein A affinity chromatography, and liquid chromatography-tandem mass spectrometry (LC-MS/MS), we found a number of novel *Gl*Actin interactors. We categorized a subgroup of these putative interactors based on their localization: marginal plate, flagella, ventral disc, nuclei, membrane, and nonspecific. Notably, no previous *Gl*Actin interactors have been localized to the marginal plate or the ventral disc, suggesting that there may be previously unappreciated roles for *Gl*Actin in these cellular structures.

## RESULTS

### Identification of noncanonical *Gl*Actin interactors.

We used a biochemical approach to identify *Gl*Actin-binding proteins, utilizing a chemical cross-linker, anti-*Gl*Actin antibodies, and protein A beads. For our purification scheme, we used buffers developed for canonical actin; whether these buffers are optimal for *Gl*Actin remains untested. Therefore, we also treated the cells with the cleavable chemical cross-linker dithiobis(succinimidyl propionate) (DSP) to ensure the stabilization of native interactions before cell lysis. This molecule reacts with both lysine side chains and the amino terminus of peptides, linking amine groups within 12 Å of one another.

**FIG 1:**
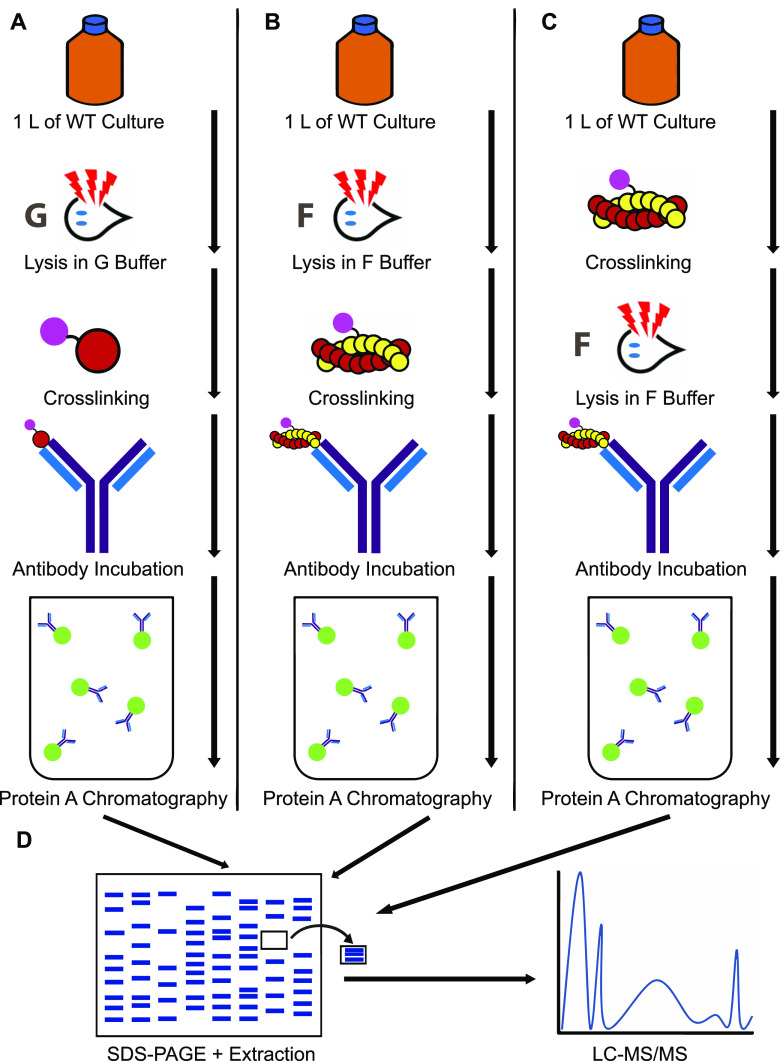
Schematic of methods. One liter (1 L) of cells was lysed under one of the following conditions: (A) in G-buffer followed by cross-linking, (B) F-buffer followed by cross-linking, or (C) treated with cross-linker and then lysed in F-buffer. Each lysate was then incubated with an anti-*Gl*Actin antibody followed by protein A chromatography. (D) The eluate was run on an SDS-PAGE gel, excised, and identified by LC-MS/MS.

To differentiate F-actin and G-actin interactors, we lysed wild-type cells under these conditions: in G-buffer, expected to favor monomeric actin, followed by DSP treatment, in F-buffer, expected to favor filamentous actin, followed by DSP treatment, or pretreated with DSP before lysis in F-buffer, expected to stabilize native interactions (Fig. 1). We then purified *Gl*Actin using an anti-*Gl*Actin antibody and protein A affinity chromatography, and subsequently identified the interactors by LC-MS/MS. Compared with low-salt G-Buffer, our F-buffer contains KCl, MgCl_2_, EGTA, and imidazole, which promote and stabilize filamentous actin (6, 11).

**FIG 2:**
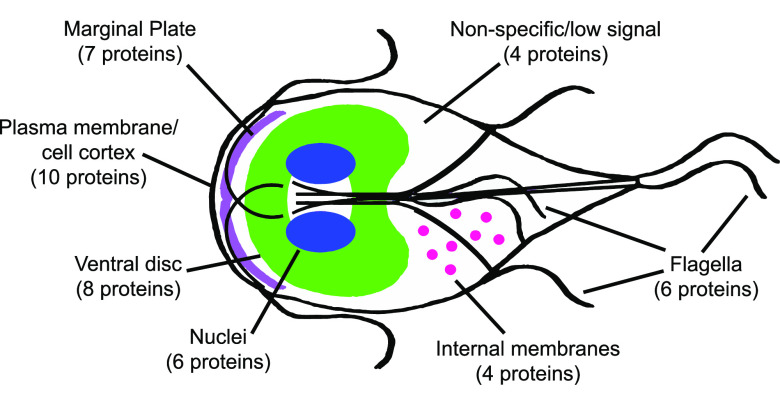
Localizations of *Gl*Actin filament interactors. Diagram of a Giardia cell, with numbers of interactors localizing to each structure in parentheses. Shown are the nuclei (blue), ventral disc (green), marginal plate (purple), flagella and axonemes (black), internal membranes, (pink), plasma membrane (black), and nonspecific/low signal.

Proteins were classified as potential F-actin interactors if they were enriched under either the DSP pretreated or F-actin buffer conditions in at least two of three replicates compared to that under the G-actin condition. Notably, proteins that had been previously identified as *Gl*Actin interactors, including 14-3-3, p28, GASP-180, and others, were also found in this screen, indicating the robustness of our methods (see Table S1 in the supplemental material). Proteins enriched under the F-actin conditions but that are commonly found as contaminants, such as ribosomal and proteasomal proteins (12), were omitted from our analysis. We also omitted metabolic proteins, as we were interested in proteins that regulated actin organization, and variant-specific surface proteins (VSPs) involved in antigenic variation (13, 14). After exclusion criteria were applied, a total of 46 proteins remained (Table 1). Many of these proteins were annotated as “hypothetical proteins,” meaning they lack homologues or known protein domains. Each candidate protein was tagged with the fluorescent protein mNeonGreen and localized in live and/or fixed cells. Five proteins had nonspecific localization or low signal (Table 1, see also Fig. S1); the others were grouped into the categories discussed below based on their localization (Fig. 2). Despite multiple attempts, we were unable to transform GL50803_21423 (beta adaptin); thus, it is absent from our analysis.

**TABLE 1:** Proteins identified as putative F-actin interactors^*a*^

ORF no.	Identity	Localization
Plasma membrane/cortical localization		
GL50803_8560	Hypothetical	Plasma membrane
GL50803_103855	VPS29	Plasma membrane
GL50803_23833	VPS35	Plasma membrane
GL50803_4259	Clathrin light chain	Plasma membrane
GL50803_8044	7 transmembrane domains	Plasma membrane and unidentified compartment
GL50803_15591	Coiled-coil protein	Plasma membrane
GL50803_14551	Alpha-6 giardin	Ventrolateral flange and plasma membrane
GL50803_17255	Hypothetical	Plasma membrane, flange, basal bodies
GL50803_5810	Hypothetical	Plasma membrane
GL50803_11684	Leucine-rich repeat protein	Plasma membrane
Internal membrane localization		
GL50803_9861	Hypothetical	Diffuse internal membrane
GL50803_13930	Arf3	Puncta
GL50803_22855	Hypothetical	Vesicles
GL50803_12999	Hypothetical	Puncta and unidentified compartment
Marginal plate localization		
GL50803_17153	Alpha-11 giardin	Marginal plate and axonemal-associated structures
GL50803_115478	Ankyrin repeat, takusan, SMC	Marginal plate and flagellar exit sites
GL50803_8854	Hypothetical	Marginal plate and flagellar exit sites
GL50803_4383	Ankyrin repeat	Marginal plate and axonemal-associated structures
GL50803_5800	Hypothetical	Marginal plate and puncta
GL50803_9030	Ankyrin repeat	Marginal plate and cytoplasmic puncta
GL50803_16522	Hypothetical	Cytoplasmic, marginal disc, nuclei
Nuclear localization		
GL50803_101212	Hypothetical	Perinuclear region
GL50803_33989	Hypothetical	Puncta and filaments within nucleus
GL50903_14299	Hypothetical	Diffuse nuclear localization with puncta
GL50803_5328	Sigma adaptin (AP-2 complex)	Diffuse nuclear localization and plasma membrane
GL50803_102813	Ankyrin repeat	Nuclear, marginal plate, cytoplasmic
GL50803_14213	Hypothetical	Diffuse nuclear localization with puncta
Flagellar localization		
GL50803_9848	Dynein light chain	Internal portions of flagella, basal bodies
GL50803_33685	Armadillo repeat	Flagella
GL50803_15097	Alpha-14 giardin	Flagella
GL50803_13774	Hypothetical	Flagellar exit sites
GL50803_4463	Dynein light chain	Flagella
GL50803_15124	Dynein light chain	Flagella
Disc localization		
GL50803_4239	Hypothetical	Ventral disc, cytoplasmic
GL50803_24451	FGF	Flagella, basal bodies, lateral crest
GL50803_8726	Hypothetical	Ventral disc, midbody
GL50803_27925	Ankyrin repeat	Ventral disc, except ventral groove
GL50803_113622	Ankyrin repeat	Ventral groove
GL50803_16844	Hypothetical	Ventral disc: ventral groove and overlap zone
GL50803_17230	Gamma-giardin	Ventral disc
GL50803_11354	Hypothetical	Flagella, lateral crest
Nonspecific localization		
GL50803_10038	Alpha-18 giardin	Diffuse nonspecific
GL50803_10429	Wos2 protein	Diffuse nonspecific
GL50803_7323	Hypothetical	Diffuse nonspecific
GL50803_6242	Translationally controlled tumor protein-like	Diffuse nonspecific
GL50803_21423	Beta-adaptin	NA

a FGF, fibroblast growth factor; NA, not available.

### *Gl*Actin interactors in the nuclei.

Giardia has two transcriptionally active nuclei, to which *Gl*Actin localizes (6). Previous studies identified conserved *Gl*Actin interactors in the nuclei, and treatments altering the phosphorylation state of *Gl*Actin affected nuclear size (10, 15). In model organisms, actin has multiple roles in the nucleus, including chromatin remodeling and regulating transcription (16).

**FIG 3:**
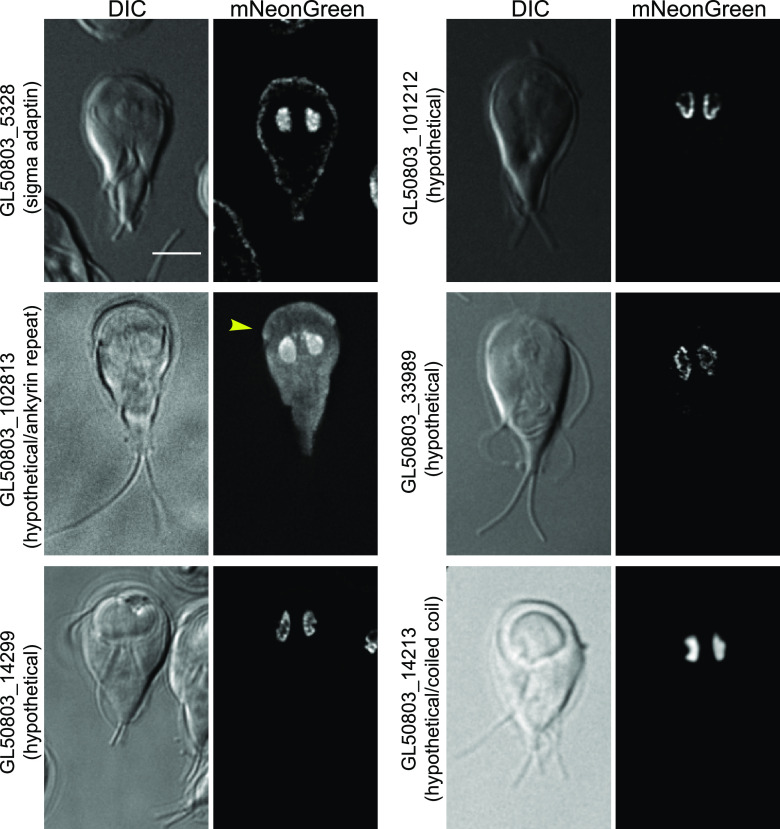
Putative *Gl*Actin interactors with nuclear enrichment. Six proteins identified were enriched in Giardia’s nuclei, based on the localization of mNeonGreen fusions. GL50803_5238 localized to the plasma membrane as well as the nuclei. GL50803_102813 was also enriched in the marginal plate (yellow arrowhead) and cytoplasm, with some puncta. GL50803_14299 and GL50803_33989 appeared closer to the nuclear envelope, while GL50803_101212 localized to the perinuclear region. GL50803_14213 was evenly distributed within the nuclei. Bar, 5 μm.

Our screen discovered six nuclear-enriched proteins, each with a distinct sublocalization (Fig. 3). GL50803_5238 (sigma adaptin, a component of the AP-2 complex) appeared in puncta within the nuclei and in association with the cell cortex. GL50803_102813 (ankyrin repeat protein), a protein upregulated during early encysation (17), also appeared in the cytoplasm, with some large puncta, as well as a slight enrichment in the marginal plate. The protein appears somewhat more concentrated toward the anterior of the nuclei. GL50803_GL14299 (hypothetical) appeared to be associated with the nuclear envelope in puncta. The perinuclear localization of GL50803_101212 (hypothetical) may represent the perinuclear endoplasmic reticulum. GL50803_33989 (hypothetical) was previously tagged with a C-terminal green fluorescent protein (GFP) tag and formed inclusion bodies in cells (18, 19), and so we tagged this protein N terminally under its native promoter. Under these conditions, GL50803_33989 localized to the nuclei, forming structures which may be filamentous. In contrast, GL50803_14213 (coiled coil domain) has no puncta or other subpattern within the nuclei.

### *Gl*Actin interactors in the flagella.

**FIG 4:**
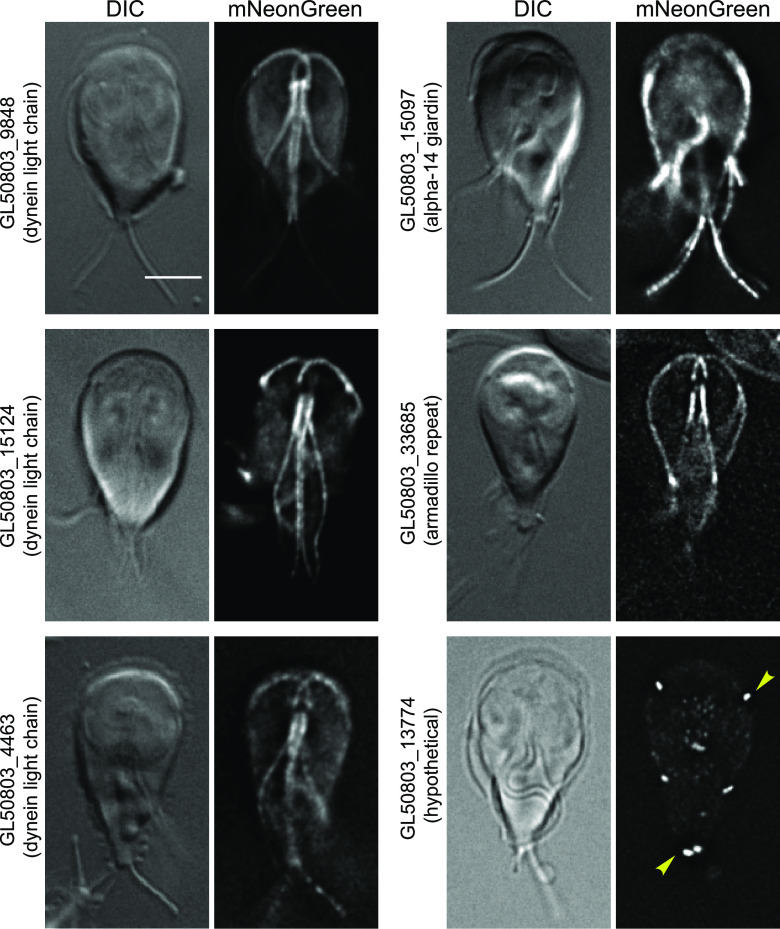
Putative *Gl*Actin interactors with flagellar localization. Six *Gl*Actin interactors localized to flagella or flagella-related structures based on the localization of mNeonGreen fusions. GL50803_15124 had a general flagellar localization. GL50803_4463 was enriched in the central axonemes, while GL50803_9848 was enriched in the basal bodies. GL50803_15097 was enriched in posterolateral, ventral, and caudal flagella. GL50803_33685 also appeared slightly in the plasma membrane. GL50803_13774 was only present in the flagellar pores (yellow arrowheads). Bar, 5 μm.

Giardia’s four sets of flagella have different cellular roles and undergo a complex developmental cycle (20, 21). Actin is a key component of flagella; in *Chlamydomonas*, six of the seven inner dynein arms form a complex with actin (22). Additionally, actin is an important regulator of intraflagellar transport and the regulation of flagellum length (23). Anti-*Gl*Actin immunostaining showed its clear enrichment within the flagella and as a helix that surrounds the caudal flagella, similar to the actin organization of the sperm midpiece (24). Our previous study identified six axonemal dynein heavy chains in addition to p28 as *Gl*Actin interactors (10). Thus, finding six flagellar-enriched proteins in this screen is consistent with previous knowledge (Fig. 4). Three of these candidate *Gl*Actin interactors are dynein light chains; while all appeared in the cytoplasm in low levels, each had their own unique flagellar enrichment pattern. GL50803_9848 appeared primarily associated with the cytoplasmic portions of axonemes, while GL50803_15124 was enriched in the entirety of the flagellar axonemes. GL50803_4463 had similar axonemal enrichment and additionally appeared in patches throughout the cell in low levels. GL50803_15097 (alpha-14 giardin) enrichment was less intense in the anterior flagella than in the other three flagellar pairs. GL50803_33685 (armadillo repeat) appeared throughout the flagella but was particularly enriched in the axonemes. In contrast, GL50803_13774 (hypothetical) appeared only in the flagellar pores, the structures where the flagella exit the cell.

### *Gl*Actin interactors in the marginal plate.

**FIG 5:**
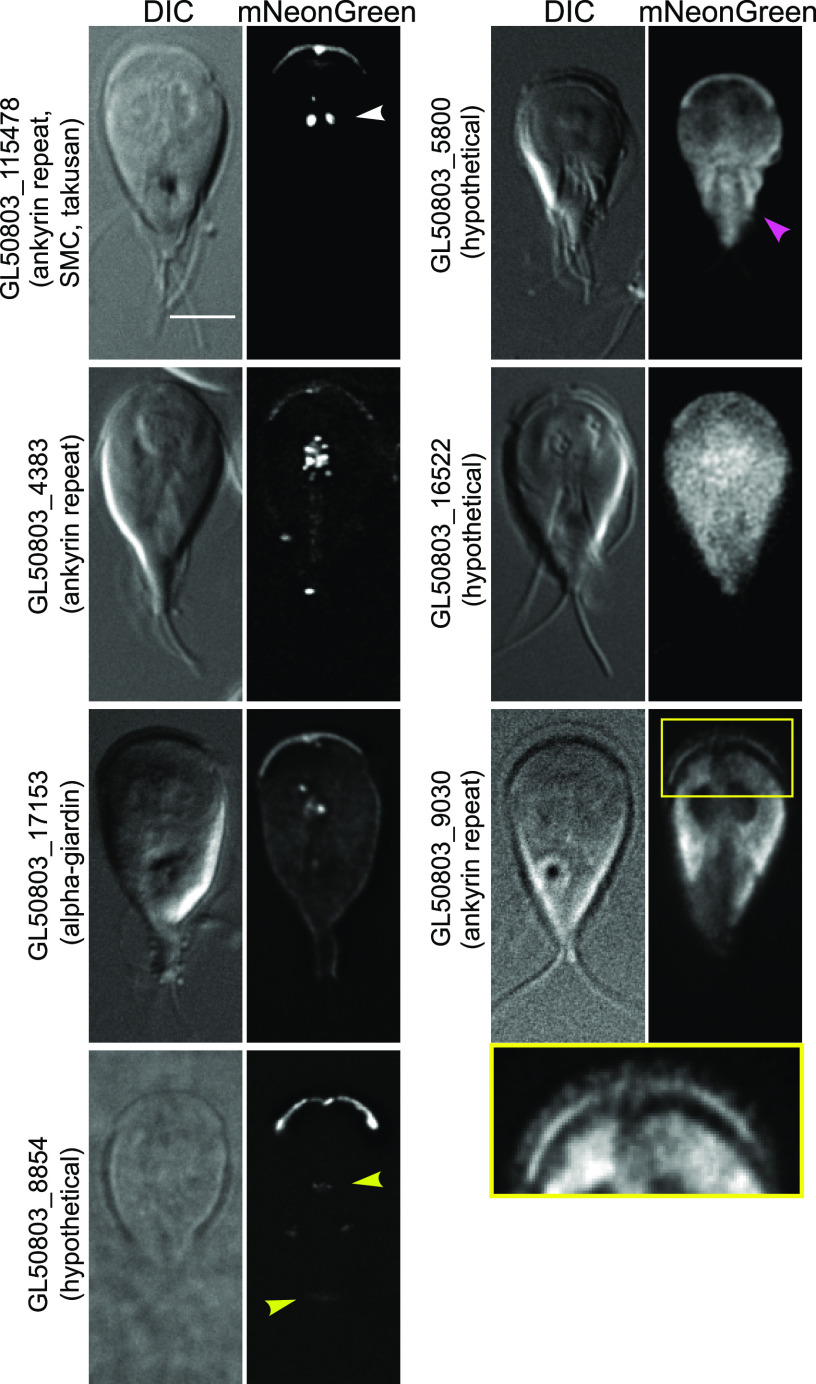
Putative *Gl*Actin interactors with marginal plate localization. Seven *Gl*Actin interactors localized to the marginal plate based on the localization of mNeonGreen fusions. GL50803_115478, GL50803_17153, and GL50803_4383 also appeared in axoneme-associated structures (white arrowhead). GL50803_8854 was enriched in the flagellar pores (yellow arrowheads). GL50803_5800 also appeared in the axonemes (magenta arrowhead) and, similar to GL50803_16522, was only slightly enriched in the plate. GL50803_9030 was additionally observed in linear arrays that may be filaments at the anterior ventrolateral flange (yellow box is magnified view) and also localized throughout the cytoplasm with enrichment in the lateral shield, but was excluded from the nuclei. Bar, 5 μm.

Seven proteins in our screen were enriched in the marginal plate (Fig. 5), a lattice-like structure at the anterior of the cell which is associated with the axonemes of the anterior flagella (25). Ultrahigh-resolution scanning electron microscopy has shown flexible filaments at the plate (26, 27), and our immunofluorescence revealed enriched *Gl*Actin there (10, 27). Little else is known about the function or composition of the marginal plate; it has been suggested to have a role in attachment, but this has remained an unexplored area of research (6, 8, 28).

In addition to their presence in the marginal plate, GL50803_115478 (ankyrin repeat), GL50803_4383 (ankyrin repeat protein), and GL50803_17153 (alpha-11 giardin) also appeared as puncta or linear stretches near the axonemes, a localization reminiscent of the mitochondrion-like mitosomes (29, 30). These puncta were not present in all cells and were variable in number (see Fig. S2). We observed 58.33% (*n* = 48) of GL50803_115478 cells contained puncta, which ranged from 1 to 3, whereas 34.6% (*n* = 52) of GL50803_17153 cells contained puncta, ranging from 1 to 4. All GL50803_4383 cells (*n* = 50) contained puncta, which ranged from 2 to 5.

Notably, multiple marginal plate proteins also appeared in microtubule-based structures. For instance, GL50803_17153 (alpha-11 giardin) appeared faintly in the disc (Fig. S2), GL50803_8854 (hypothetical) also localized to the flagellar pores, and GL50803_5800 (hypothetical) was slightly enriched in the axonemes.

Although enriched in the marginal plate, GL50803_5800, GL50803_16522, and GL50803_9030 (ankyrin repeat) were also distributed throughout the cytoplasm; GL50803_16522 was also slightly enriched in the nuclei. GL50803_9030 also appeared to be more concentrated in the anterior of the cell in the ventrolateral flange and in the lateral shield, the ventral region of the plasma membrane. Both of these structures contact the surface of substrates during attachment (31).

### *Gl*Actin interactors in internal membranes.

Although Giardia lacks many organelles, including the Golgi apparatus, lysosomes, and peroxisomes, it still has a defined endomembrane system. *Gl*Actin is important for receptor-mediated endocytosis, membrane remodeling during abscission, and the trafficking of cyst wall protein during encystation (6, 8, 32).

**FIG 6:**
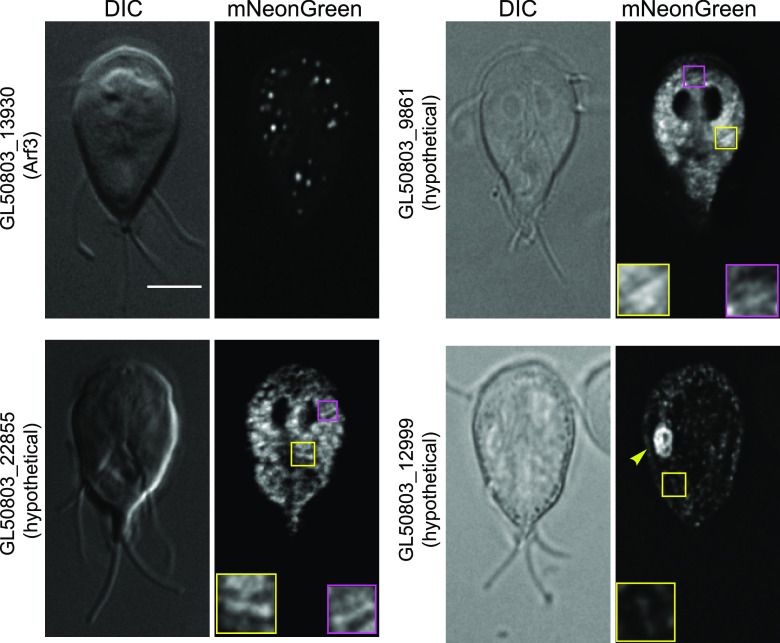
Putative *Gl*Actin interactors in the endomembrane system. Four *Gl*Actin interactors localized to internal structures likely to be the endomembrane system. GL50803_13930 was concentrated in a few stable puncta, while GL50803_22855 and GL50803_9861 were widely distributed throughout the cytoplasm. GL50803_12999 localized to cytoplasmic puncta and a large oblong organelle (yellow arrowhead). Except for Arf3, these proteins are sometimes seen in linear arrays (shown in insets) that could also indicate a connection to actin filaments. Bar, 5 μm.

Four of our novel *Gl*Actin interactors have localization consistent with that of membrane-bound compartments (Fig. 6). Apart from the previously mentioned GL50803_101212, which may be in the perinuclear endoplasmic reticulum (ER), two proteins localized to internal structures which we suspect to be the endoplasmic reticulum: GL50803_13930 (Arf3) and GL50803_22855 (hypothetical). GL50803_22855 (hypothetical) appeared to be localized throughout the entirety of the organelle, whereas the puncta of GL50803_13930 (Arf3) are consistent with the ER exit sites (33). GL50803_9861 (hypothetical) had a punctate localization which could represent small vesicles. Intriguingly, GL50803_12999 (hypothetical) localized to a previously undescribed organelle as well as puncta that could be peripheral vacuoles and the ER.

### *Gl*Actin interactors at the cortex and plasma membrane.

**FIG 7:**
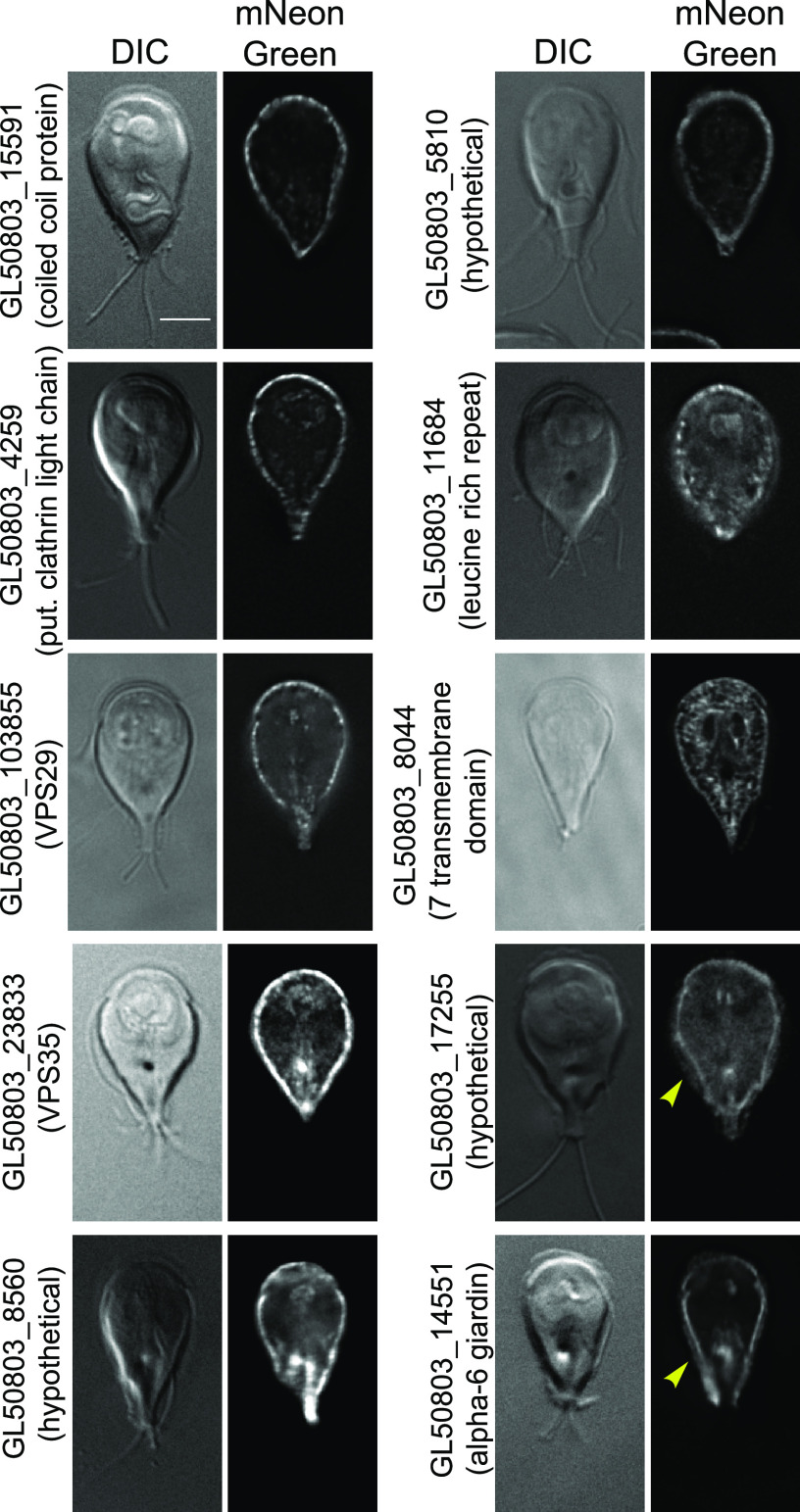
Putative *Gl*Actin interactors with cortical or plasma membrane localization. Ten *Gl*Actin interactors localized to the cell cortex based on the localization of mNeonGreen fusions. Clathrin light chain GL50803_4259 is expected at the cell cortex. GL50803_5810 also had a slight localization to the nuclear envelope, and GL50803_103855, GL50803_23833, and GL50803_8560 had a slightly punctate distribution within the plasma membrane. GL50803_15591 and GL50803_11684 were even more punctate, with individual puncta clearly visible. GL50803_8044 localized to the ER. Both GL50803_14551 and GL50803_17255 extended into the ventrolateral flange (yellow arrowheads); the latter was also enriched in the basal bodies. Bar, 5 μm.

We found 10 putative interactors which localized to the cell cortex, composing the largest group in the screen. This is consistent with *Gl*Actin and actin in general having roles in regulating cell shape, cell polarity, and membrane trafficking (Fig. 7). Despite a shared generalized localization, each putative interactor had its own individual pattern. GL50803_15591 (coiled-coil protein), GL50803_4259 (clathrin light chain), GL50803_103855 (VPS29), GL50803_23833 (VPS35), GL50803_8560 (hypothetical), GL50803_5810 (hypothetical), and GL50803_11684 (leucine-rich repeat protein) all had a punctate localization at the cell cortex, likely in association with the plasma membrane. The internal membrane localization of GL50803_8044 (7 transmembrane domains) localized to both the plasma membrane and what is likely the ER, as transmembrane proteins are necessarily trafficked through this organelle. Both GL50803_14551 (alpha-6 giardin) and GL50803_17255 (hypothetical) were present in the ventrolateral flange, a thin membranous structure that protrudes from the plasma membrane and contributes to parasite attachment (34, 35).

### *Gl*Actin interactors in the ventral disc.

The ventral adhesive disc is a microtubule-based structure unique to Giardia, which allows cells to attach to the small intestine of their host or to the side of a culture tube *in vitro*. The dome-shaped disc is composed of roughly 100 microtubules which form a stable sheet that wraps into a spiral and overlaps itself (36). In the center of the disc is the bare area, a region devoid of microtubules but containing membrane-bound vesicles involved in membrane trafficking (31).

Eight putative *Gl*Actin interactors are enriched in the disc, representing the second largest group of putative interactors. GL50803_8726, GL50803_16844, GL50803_4239, and GL50803_17230 had not been localized when we started this work but have since been identified as disc-associated proteins (DAPs) (19); they are included here for the sake of completeness. GL50803_27925, GL50803_113622, GL50803_24451, and GL50803_11354 are new DAPs.

**FIG 8:**
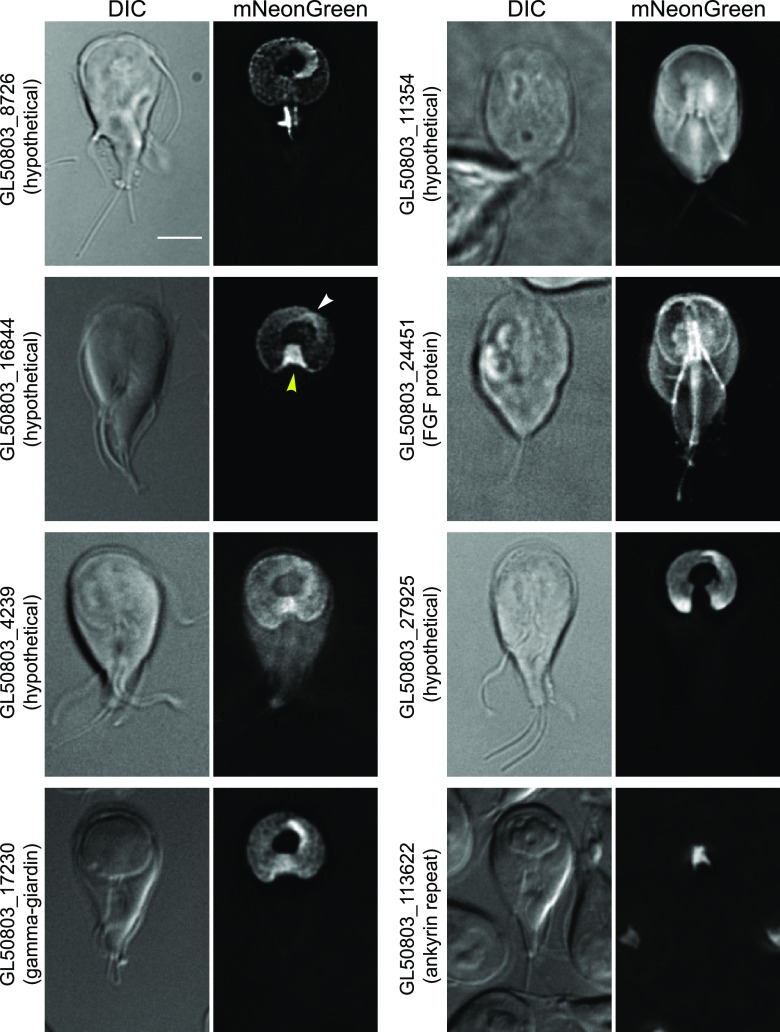
Putative *Gl*Actin interactors with ventral disc localization. Eight proteins identified localized to the ventral disc based on mNeonGreen fusions. GL50803_8726 was more prominent on the disc margin and the overlap zone and appeared on the median body. GL50803_16844 was enriched in the ventral groove (yellow arrowhead) and the overlap zone (white arrowhead); GL50803_17230 was similarly enriched in the overlap zone. GL50803_4239 had a slightly brighter signal in the ventral groove, the only place where GL50803_113622 localized. GL50803_11354 and GL50803_24451 faintly outlined both the ventral disc and the flagella. GL50803_27925 is excluded from the ventral groove. Bar, 5 μm.

The localizations within the ventral disc varied widely (Fig. 8). Four of these proteins have no recognized domains, including GL50803_8726 (hypothetical), which localized to the entirety of the ventral disc in addition to the median body, a reservoir of microtubules supporting rapid disc assembly during mitosis (7). GL50803_16844 (hypothetical), GL50803_4239 (hypothetical), and GL50803_1720 (gamma-giardin) were also apparent in the entirety of the disc but enriched in various regions within it. Both GL50803_11354 (hypothetical) and GL50803_24451 (FGF domain) had less-distinct disc enrichment. The remaining two disc-localized proteins have ankyrin-repeat domains and, coincidentally, had inverse patterns of localization: while GL50803_27925 appeared everywhere in the disc except the ventral groove, GL50803_113622 localized to only the ventral groove.

### Colocalization of candidates that interact with *Gl*Actin.

**FIG 9:**
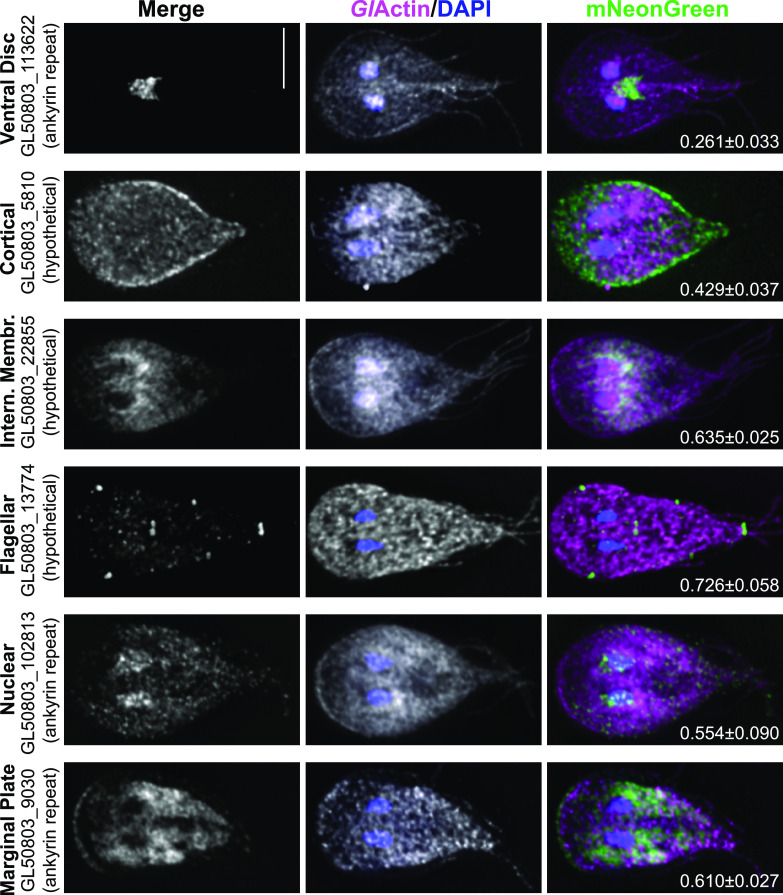
Immunostained *Gl*Actin is localized throughout the cell. One protein from each category of localization colocalized with *Gl*Actin in fixed Giardia trophozoites. Nuclei were stained with 4′,6-diamidino-2-phenylindole (DAPI; blue), *Gl*Actin is magenta, and each mNeonGreen-tagged interactor is in green. Average projections are shown. Bar, 5 μm. Pearson’s correlation coefficients using Costes randomization were calculated for three images of each protein, shown as the mean ± standard deviation (SD) on each merge. The Costes *P*-value was 1 for all candidates.

As there is no *Gl*Actin marker available for live imaging, we performed immunofluorescence assays on one protein per category of localization with an anti-*Gl*Actin antibody to colocalize them (Fig. 9). Phalloidin does not bind to *Gl*Actin (6), and so polymerized *Gl*Actin is difficult to distinguish from pools of monomeric *Gl*Actin in fixed cells. Similarly, because *Gl*Actin is ubiquitously distributed throughout the cell, colocalization is not easily discerned. We used the JACoP plugin from ImageJ to evaluate colocalization. Based on Pearson’s correlation coefficients (PCCs), the most prominent colocalization between *Gl*Actin and its putative interactors can be seen in the internal membrane protein GL50803_22855 and GL50803_13774, which localizes to the flagellar pores. The nuclear-enriched GL50803_102813 and the marginal plate protein GL50803_9030 also had PCCs of >0.5, indicating colocalization.

**FIG 10:**
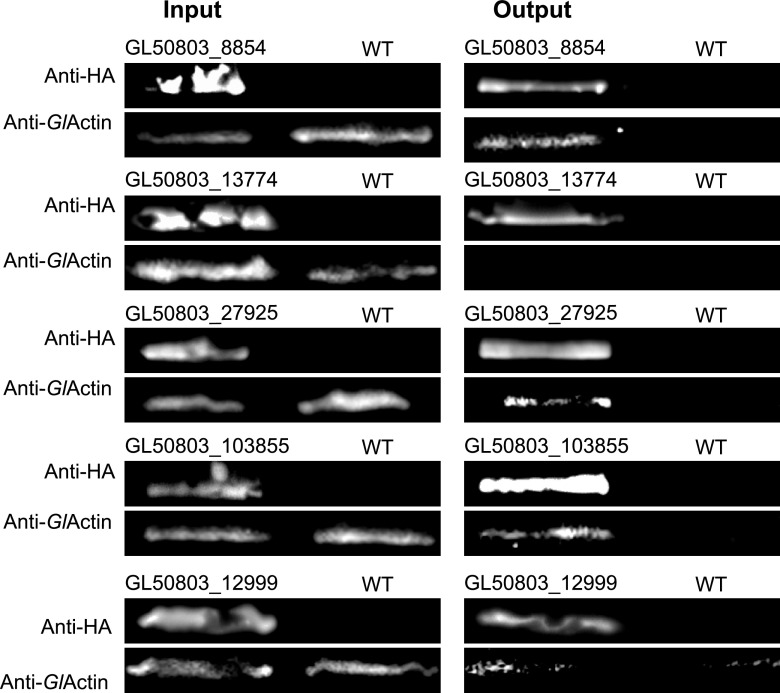
Reciprocal co-immunoprecipitation. One protein from each category of localization was tagged with 3×HA and immunoprecipitated, followed by anti-*Gl*Actin Western blotting. Three of the six proteins tagged definitively complexed with *Gl*Actin, including GL50803_8854 (marginal plate), Gl50803_103855 (plasma membrane), and GL50803_27925 (ventral disc). The expression of GL50803_33989 (nuclear) was too low to be visible on Western blot and is thus not shown.

To validate the association with *Gl*Actin for a subset the identified proteins, we made 3× hemagglutinin (3×HA) constructs to perform co-immunoprecipitations, following the same lysis and cross-linking procedures described above. We chose one protein per category based on its presence in all three replicates of our screen. The exception is GL50803_8854, as none of the proteins from the marginal plate category were found in all three replicates. We chose GL50803_8854 based on its high expression and presence at both the marginal plate and flagellar pores, structures where *Gl*Actin is enriched. We were able to confirm complex formation between *Gl*Actin and GL50803_27925 (disc), GL50803_8854 (marginal plate), and VPS29 (plasma membrane). Association between *Gl*Actin and GL50803_12999 (internal membranes) was ambiguous (Fig. 10), possibly due to the latter’s association with membranes and/or low expression. The nuclear interactor GL50803_33989 did not express at high enough levels to be visible on a Western blot (data not shown). Our co-immunoprecipitation results confirm that we have identified *Gl*Actin-associated proteins, increasing our confidence in the data presented here. It should be noted, however, that despite its colocalization with *Gl*Actin, the flagellar pore protein GL50803_13774 did not co-immunoprecipitate with *Gl*Actin. This does not necessary indicate that they do not interact; the 3×HA tag could have interfered with the interaction. Another possibility is that the interaction is too weak to be detected by Western blotting, since LC-MS/MS is a more sensitive technique.

## DISCUSSION

We performed LC-MS/MS to identify putative *Gl*Actin interactors and found them to localize to multiple regions within the cell, including the nuclei, plasma membrane, endomembrane system, marginal plate, flagella, and ventral disc. We also found a number of previously validated *Gl*Actin-binding proteins (see Table S1 in the supplemental material), as well as several proteins which had already been localized in live cells by the Dawson lab (UC Davis) as part of the Giardia genome database project (see Table S2) (19). Seventeen of the 46 proteins revealed by this screen have no recognizable identity and lack conserved domains; thus, we were unable to find any clues to their function within their sequence. We also used I-TASSER to search for structural homologs, but this search, too, yielded no results (35). Six proteins identified in our screen contain ankyrin repeat domains, scaffolds for protein recognition and interaction, which are present in actin regulators in other organisms (37, 38). We also found four alpha-giardins, annexin-like cytoskeletal proteins unique to Giardia (39). The membrane-localized alpha-6 giardin may have a role in membrane organization, similar to that of the canonical annexin A-II, which binds F-actin and mediates its interactions with membranes (40, 41).

Nuclear actin has been described in other eukaryotes, including mammals, plants, insects, and other protozoa (42). Five of our putative F-actin interactors localized to Giardia’s two nuclei. When immunostained, *Gl*Actin is visible in the nuclei; our lab previously demonstrated roles for *Gl*Actin in nuclear positioning and size (6, 10, 15).

Both monomeric and filamentous actin have functions within the nucleus. G-actin has a role in gene regulation through formation of the preinitiation complex and in chromatin remodeling through its association with the BAF complex (42, 43). In contrast, F-actin has a role in intranuclear mobility, WNT-dependent transcription, and nuclear stability (16, 42, 44). Notably, most of these functions depend on nuclear myosins, which are missing from the Giardia genome (3); however, it is still possible that *Gl*Actin fulfills these functions, particularly since a number of our putative interactors localize to the nuclei.

In model organisms, actin also works not only within but immediately around the nucleus for protection as well as anchoring the nucleus and in nuclear positioning during mitosis, mediated by the linker of nucleoskeleton and cytoskeleton (LINC) complex (44, 45). While Giardia lacks the LINC complex (6), it is likely that actin is still involved in nuclear positioning, since *Gl*Actin knockdown results in mislocalized nuclei (6).

In model eukaryotes, the Arp2/3 complex drives actin polymerization to mediate clathrin-coated pit internalization, which is nucleated by the AP-2 complex (46). Although *Gl*Actin’s role in membrane trafficking has been demonstrated, its possible involvement in clathrin-mediated endocytosis remains controversial (47, 48). *Gl*Actin was not identified as an interactor of clathrin heavy chain in an earlier study (49), but other evidence points to their interaction (50). Furthermore, depletion of *Gl*Actin by morpholinos results in decreased uptake of BODIPY-LDL, which is mediated by clathrin (6, 51). The presence of the putative clathrin light chain among our interactors points to some involvement. Due to the absence of most conserved endocytic proteins, the mechanism for clathrin-mediated endocytosis in Giardia is poorly understood. The clathrin heavy chain has been demonstrated to interact with components of the AP-2 adaptor complex, specifically, alpha- and beta-adaptins, which localize to the plasma membrane (49). The mu subunit of AP-2 has been localized to the hybrid endosome/lysosome peripheral vacuoles and plasma membrane (51). It was therefore unexpected to see sigma adaptin, central to the structure of the AP-2 complex (52–54), localize to the nuclei in addition to the plasma membrane. However, some of the other adaptins, notably, alpha adaptin, have a function in nuclear translocation (55); it is possible that both sigma adaptin and *Gl*Actin are involved in this process as well, particularly since sigma adaptin has both nuclear and plasma membrane localization.

Two other membrane-localized proteins, vacuolar protein sorting 29 (VPS29) and VPS35, are subunits of the cargo recognition particle of the retromer complex. Retromer is involved in the endosome-to-Golgi apparatus retrograde transport pathway and mediates the recycling of membrane receptors in yeast and mammals (56). Previous work in Giardia, which shows VPS29 localizing to the ER, conflicts with our results; however, the previous study was performed in fixed cells, and fixation/permeabilization can sometimes disrupt membrane protein localization (57, 58). However, the same study localized VPS35 to the plasma membrane (59), and so it is expected that at least a subset of VPS29 would also localize to the plasma membrane. While VPS26, the other subunit of the cargo recognition particle, did not fit our criteria for possible F-*Gl*Actin interactors, it did appear in our screen (see Table S3). Since there is no Golgi apparatus in Giardia and the sorting nexins which constitute the other retromer subunit are also missing from the Giardia genome, the role of Giardia’s cargo recognition particle is unknown. VPS35 has been shown to bind to a hydrolase receptor, but its function in doing so was not explored (59). Furthermore, while the canonical VPS35 is known to coordinate with actin through interaction with FAM21 and the WASH complex (60), these are also absent *in* Giardia.

While the Giardia genome lacks moesin, ezrin, radixin, or any ERM domain proteins, *Gl*Actin does localize to the cellular cortex, and so one or more of the putative interactors also localized here could fulfill the role of a cortical cross-linker. Furthermore, *Gl*Actin-depleted cells have defects in cell polarity and cell shape (6). Another possible role for *Gl*Actin interactors localized to the plasma membrane is in phagocytosis; evidence for this process taking place in Giardia has recently been published (61). If further research shows Giardia to be capable of phagocytosis, *Gl*Actin is likely to be involved (61, 62), given its role in phagocytosis in other organisms.

A notable pattern in two cortical proteins, the alpha-giardin GL50803_14551 and the hypothetical protein GL50803_17255, was their reach into the ventrolateral flange (VLF). This structure is a membrane reservoir that skirts Giardia cells and contributes to attachment (34, 35). Short *Gl*Actin filaments appear in the VLF, and another novel actin interactor, flangin, has been implicated in its function (34); it is likely that more of these interactors are important for the maintenance and function of the VLF.

Many of our newly identified interactors localize to axonemes. Actin is a key component of the flagellum inner dynein arm, although the actin within the inner arm is monomeric and complexed with a p28 homodimer (63); additionally, actin appears to play a structural role in the gamma-tubulin ring complex (γ-TuRC) (64). Immunostaining indicates *Gl*Actin to be within the flagella, and we previously identified p28 as an interactor (22, 65). Actin’s role in the flagella of other organisms is not merely structural; for instance, actin polymerization is required for flagellar biogenesis and intraflagellar transport in Chlamydomonas reinhardtii (23, 66). Morpholino knockdown of *Gl*Actin results in flagella which are mispositioned or even missing, implicating *Gl*Actin in flagellar positioning (6). The two flagellar pore interactors we identified could also be involved in this process; future studies could investigate the role of these proteins through depletion to test if resulting flagella lack stability, are trapped within the cell body, or exit the cell at an incorrect location.

Despite the reduction of Giardia’s endomembrane system, it has a relatively conventional ER, and two of our proteins had ER-like localizations, consistent with *Gl*Actin’s previously established role in membrane trafficking. Two others, GL50803_12999 (hypothetical) and GL50803_8044 (7 transmembrane domain), localized to an undescribed compartment. Since Giardia has unique organelles such as the Golgi-like encystation specific vesicles, it is likely that more compartments exist in Giardia than are currently recognized, and these proteins may be involved in their function.

The marginal plate, a crescent-shaped structure at the anterior of the cell, has not yet been functionally studied, limiting our ability to infer the function of the proteins located there. While it has been suggested to be involved in attachment, this role is speculative (67). Since immunofluorescence assays show enrichment of *Gl*Actin at this site, it is possible that the meshwork of filaments seen in electron micrographs (67) are composed of *Gl*Actin or that *Gl*Actin acts upon this structure. Intriguingly, after depletion, *Gl*Actin localization persists at the marginal plate, suggesting that the associated *Gl*Actin is highly stabilized (15).

Of all the structures to which these putative *Gl*Actin interactors localize, the ventral disc is perhaps the most surprising. One previous study localized *Gl*Actin to the ventral disc (68); however, this study used a heterologous antibody, raised against chicken gizzard actin, for immunostaining. This finding is very likely artifactual, given that the same study found similar localization patterns for actin-binding proteins not present in the Giardia proteome (68, 69). While the disc itself is composed of microtubules, their polymerization dynamics are not responsible for flexion and/or attachment; it is possible that *Gl*Actin, as a force-generating protein, plays a role in this process. Giardia’s ability to attach, and thus its health, depends on the disc, and as this structure is unique to Giardia, many of the proteins that localize there have no known relationship to proteins in other organisms. It is noteworthy that the different disc proteins had different localizations, indicating that the different regions of the disc may have different relationships to *Gl*Actin. Perhaps most notable are GL50803_16844 and GL50803_113644, as they are highly enriched in the margin near the overlap zone and/or the ventral groove; these regions are hypothesized to regulate fluid flow beneath the ventral disc (70). While *Gl*Actin does not appear concentrated in the ventral disc in immunofluorescence images, this could be due to steric hindrance between the antibody and proteins on the ventral disc, similar to the faint tubulin immunostaining seen in the disc compared to the amount of signal observed when using fluorescently tagged tubulin (7). Another possibility is that F-actin is localized to the disc but it is obscured by the pool of monomeric *Gl*Actin in our immunostaining. Defects in one of our disc-localized interactors, gamma-giardin, results in misshapen discs but normal adherence, suggesting the role of gamma-giardin to be structural rather than actively mechanical (71). Notably, over half of our interactors with previously reported localizations were at the ventral disc (Table S2) (19, 72).

Here, we have characterized a number of putative *Gl*Actin interactors, with a diversity of localizations and possible applications. Study of *Gl*Actin is challenging, as we still lack an actin reporter for live imaging in Giardia, and markers that generally recognize actin in other eukaryotes, such as LifeAct, F-tractin, and Utrophin, do not label filamentous structures in Giardia. The actin-binding domains of the proteins identified in our study could be mapped and potentially used as *in vivo Gl*Actin markers. Of particular interest are the proteins which localize to the ventral disc, the ventrolateral flange, and the marginal plate. If, as hypothesized, the plate is important for attachment, our work has potentially uncovered a connection between *Gl*Actin and all of Giardia’s mechanisms for attachment, which is required for maintaining infection. Thus, there are ample opportunities for drug development in further study of these proteins, particularly since few of them have homologues in humans, thereby reducing the likelihood of unwanted effects.

## MATERIALS AND METHODS

### Parasite strain and growth conditions.

G. lamblia strain WB clone 6 (ATCC 50803; American Type Culture Collection) was cultured under standard conditions (73).

### Lysis and cross-linking.

A 3-liter culture of wild-type Giardia trophozoites was grown to confluence in 1-liter bottles as described previously (10). Cells were iced for 2 h to induce detachment, centrifuged for 30 min at 1,500 × *g* at 4°C, and washed twice with 1× HEPES-buffered saline (HBS) plus 1× HALT protease inhibitor cocktail (Thermo Scientific catalog number 78430); this was followed by resuspension and centrifugation for 15 min at 1,500 × *g* at 4°C. Each culture was then resuspended in 1.6 ml of buffer. Two were resuspended in F-buffer (20 mM HEPES, 0.2 mM CaCl2, 80 mM KCl, 10 mM imidazole, 1 mM MgCl_2_, 1 mM EGTA, 5% glycerol, 10 mM ATP, 1× HALT protease inhibitors, pH 7.2), while one was resuspended in G-buffer (20 mM HEPES, 0.2 mM CaCl2, 0.2 mM ATP, 1× HALT protease inhibitors, pH 7.2).

One of the F-buffer cultures was then treated with cross-linker DSP [dithiobis(succinimidyl propionate)] (Thermo Scientific catalog number 22585) at a final concentration of 1 mM, incubated for 30 min at room temperature, quenched with Tris-HCl (pH 7.5) to a final concentration of 20 mM, incubated for 15 min, and then lysed in the manner described below. The other F-buffer culture and the G-buffer cultures were lysed by sonication (pulse for 25 s each at 20% power with 1-min rest between each sonication), repeated a total of 4 times minimum. Sonicated cells were allowed to rest on ice for 30 min and then cleared with a 10,000 × *g* spin for 10 min at 4°C. The supernatant of these two cultures was then treated with DSP cross-linker at a final concentration of 1 mM, incubated for 30 min at room temperature, quenched with Tris-HCl (pH 7.5) to a final concentration of 20 mM, and incubated for 15 min. This was repeated for a total of three independent replicates per condition.

### Purification and sample preparation for mass spectrometry.

Lysates were incubated overnight with an anti-*Gl*Actin antibody (6) at 4°C and then incubated with 500 μl Pierce protein A agarose (catalog number 20333) bead slurry and incubated with end-to-end mixing for 2 h at room temperature. A total of four washes were performed by adding 0.5 ml of wash buffer (G- or F-buffer with an additional 150 mM NaCl), vortexing, and centrifuging for 2 to 3 min at 2,500 × *g*. For elution, 250 μl of 0.2 M glycine (pH 2.5) was added to the beads and incubated for 5 min, the sample was centrifuged for 3 min at 2,500 × *g*, and the supernatant was collected. This step was then repeated, and the two eluate fractions were combined. The pH was then neutralized through addition of 50 μl 1 M Tris-HCl (pH 7.5).

For each condition, 5× sample buffer (0.225 M Tris-HCl, pH 6.8, 50% glycerol, 5% SDS, 0.05% bromophenol blue, 0.25 M dithiothreitol [DTT]) was added to a final concentration of 1× subsequent to running on a 10% SDS-PAGE gel and stained with SYPRO Ruby (Pierce). Bands corresponding to the heavy and light antibody chains were excised from the gel and discarded; the remainder of the gels were divided into rectangles of approximately equal size. These rectangles were cut into squares of approximately 1 to 2 mm, washed with 200 μl water, and then incubated with 200 μl of 50% acetonitrile and 25 mM ammonium bicarbonate for 5 min. After addition of another 200 μl acetonitrile and further incubation for 1 min, the gel was dried with a speed vacuum, followed by another addition of 50 μl of 25 mM ammonium bicarbonate and 10 mM Tris(2-carboxyethyl)phosphine hydrochloride (TCEP). Samples were then incubated at 60°C for 1 h, after which, the supernatant was removed, replaced with 50 μl of 25 mM ammonium bicarbonate and 10 mM iodoacetamide, and then incubated for 20 min in the dark. Another wash was performed with 400 μl water, and the 5-min incubation with 200 μl 50% acetonitrile and 25 mM ammonium bicarbonate and following steps were repeated. Protein samples were then digested with 20 μl of 0.01 μg/μL Promega trypsin in 25 mM ammonium bicarbonate. More ammonium bicarbonate was added to cover, followed by overnight digestion. Supernatant was then removed for LC-MS/MS analysis. Protein was further extracted from the gels by addition of 50 μl of acetonitrile, vortexing, and removing and saving the supernatant. One last extraction was performed by addition of 50 μl 60% acetonitrile and 0.1% formic acid, vortexing for 5 min, and then removing the supernatant. The supernatants were then combined, dried, and resuspended in solvent A and then analyzed by LC-MS/MS as described below.

### Liquid chromatography and mass spectrometry.

Liquid chromatography-mass spectrometry was performed on a Velos Pro (Thermo) with an EasyLC 1000 high-performance liquid chromatography (HPLC) and autosampler system (Thermo). Samples were solubilized in loading buffer (0.1% trifluoroacetic acid and 2% acetonitrile in water), and 6 μl was injected via the autosampler onto a 150-μm Kasil fritted trap packed with Reprosil-Pur C_18_-AQ (3-μm bead diameter; Dr. Maisch) to a bed length of 2 cm at a flow rate of 2 μl/min. After loading and desalting using a total volume of 8 μl of loading buffer, the trap was brought on-line with a pulled fused-silica capillary tip (75-μm inside diameter [i.d.]) packed to a length of 25 cm with the same Dr. Maisch beads. Peptides were eluted off the column using a gradient of 2% to 35% acetonitrile in 0.1% formic acid over 90 min, followed by 35% to 60% acetonitrile over 5 min at a flow rate of 250 nl/min. The mass spectrometer was operated using electrospray ionization (2 kV) with the heated transfer tube at 200°C using data-dependent acquisition (DDA), whereby a mass spectrum (*m/z* 400 to 1600, normal scan rate) was acquired with up to 15 MS/MS spectra (rapid scan rate) of the most intense precursors found in the MS1 scan.

Database searches were performed using Comet (74) searched against the protein sequence database GiardiaDB 3.1_GintestinalisAssemblageA_AnnotatedProteins.fasta to which was appended sequences of common contaminants (e.g., human keratins). The peptide mass tolerance was 3 Da, and the fragment ions were sorted into 1-Da bins (roughly corresponding to a fragment ion mass tolerance of ±0.5 Da). Semitryptic cleavages and up to two missed tryptic cleavage sites were allowed. Oxidized methionine and acetylation of the protein N termini were allowed as variable modifications. Carbamidomethylated cysteine was a fixed modification. False discovery rates (1%) were determined using PeptideProphet (75), and protein inferences were made using ProteinProphet (76). Spectral count analysis was performed using Abacus (77).

### Bioinformatics.

Delta-BLAST, Protein BLAST, and Pfam searches were used to identify homologues and conserved domains for each putative interactor. I-TASSER was used to search for structure-based function prediction.

### Vector construction.

All constructs in this study were C-terminal fusions, with the exception of the N-terminally tagged GL50803_33989 and GL50803_7323, using Gibson reactions with linearized vectors and PCR products (7, 78). For primer sequences and workflow, see Table S4 in the supplemental material.

### Immunofluorescence microscopy.

Fixation and imaging were performed as described in previous work (32). FIJI/ImageJ was used for image analysis, including colocalization analysis by Just Another Colocalization Plugin (JACoP) (79).

### Live cell imaging.

Cells were chilled with ice for 15 min to detach them from the culture tube and then placed into an Attofluor cell chamber (Molecular Probes) and incubated in a GasPak EZ anaerobic pouch (BD) or a Tri-gas incubator (Panasonic) set to 2.5% O_2_, 5% CO_2_ for 90 min at 37°C. Cells were then washed four times with HEPES-buffered saline (137 mM NaCl, 5 mM KCl, 0.91 mM Na_2_HPO_4_-heptahydrate, 5.55 mM glucose, 20 mM HEPES, pH 7), overlaid with a mixture of 0.7% ultralow gelling agarose (Sigma A2576) melted in HEPES-buffered saline, cooled for 10 min at room temperature to solidify, and then imaged. Live cell imaging was performed on a DeltaVision Elite microscope (GE) equipped with differential interference contrast (DIC) optics, using a 100× 1.4 numerical aperture (NA) or 60× 1.42 NA lens objective and a scientific complementary metal oxide semiconductor (sCMOS) 5.4 PCle air-cooled camera (PCO-TECH).

### Co-immunoprecipitation.

Five hundred milliliters of Giardia cell cultures were grown for 3 days and then iced for 2 h to detach and spun at 1,500 × *g* at 4°C. Cells were then washed twice in HBS with 2× HALT protease inhibitors, 10 μM chymostatin, 1 μM leupeptin, and 1 μM E64.

Each pellet was resuspended to a final volume of 1.2 ml, and 100 mM DSP in dimethyl sulfoxide (DMSO) was added to a final concentration of 1 mM and incubated at room temperature for 30 min. The reaction was quenched for 15 min with an addition of Tris-HCl (pH 7.4), final concentration 20 mM. Cells were then pelleted by spinning for 7 min at 700 × *g* and resuspended in 350 μl lysis buffer (80 mM KCl, 10 mM imidazole, 1 mM MgCl_2_, 1 mM EGTA, 5% glycerol, 20 mM HEPES, 0.2 mM, CaCl_2_, 10 mM ATP, 0.1% Triton X-100, 500 mM NaCl, pH 7.2).

Cells were then lysed by sonication and cleared as described above. A volume of 17.5 μl of equilibrated EZview Red anti-HA affinity gel (Sigma) was added to each tube of lysate and then incubated at 4°C with end-over-end mixing for 1 h. Beads were then spun at 8,200 × *g* for 30 s and the supernatant was discarded, followed by a total of three washes with 750 μl wash buffer (80 mM KCl, 10 mM imidazole, 1 mM MgCl_2_, 1 mM EGTA, 5% glycerol, 20 mM HEPES, 0.2 mM CaCl_2_, 10 mM ATP, 0.5% Tween, 500 mM NaCl, pH 7.2). Each wash consisted of end-over-end rotation for 5 min followed by a 30-s spin at 8,200 × *g*. Protein was then incubated with 50 μl of 8 M urea at room temperature (RT) for 20 min to elute, followed by the addition of 5× sample buffer (Qiagen) to a final concentration of 1×. The sample was boiled for 5 min at 98°C and run on a 12% SDS-PAGE gel, followed by Western blotting according to the protocol described previously (7).

### Data availability.

The mass spectrometry proteomics data were deposited to the ProteomeXchange (80) Consortium via the PRIDE partner repository with the data set identifier PXD026067 (81).
