# Supplementary material for: Identification of Actin Filament-Associated Proteins in Giardia lamblia
Source: Microbiol Spectr. 2021 Jul 21;9(1):10.1128/spectrum.00558-21. doi: 10.1128/spectrum.00558-21 (PMC8552679; doi:10.1128/spectrum.00558-21)
Supplement: SUPPLEMENTAL FILE 5 — Supplemental material. Download SPECTRUM00558-21_Supp_5_seq5.pdf, PDF file, 1.1 MB. [file spectrum00558-21_supp_5_seq5.pdf]

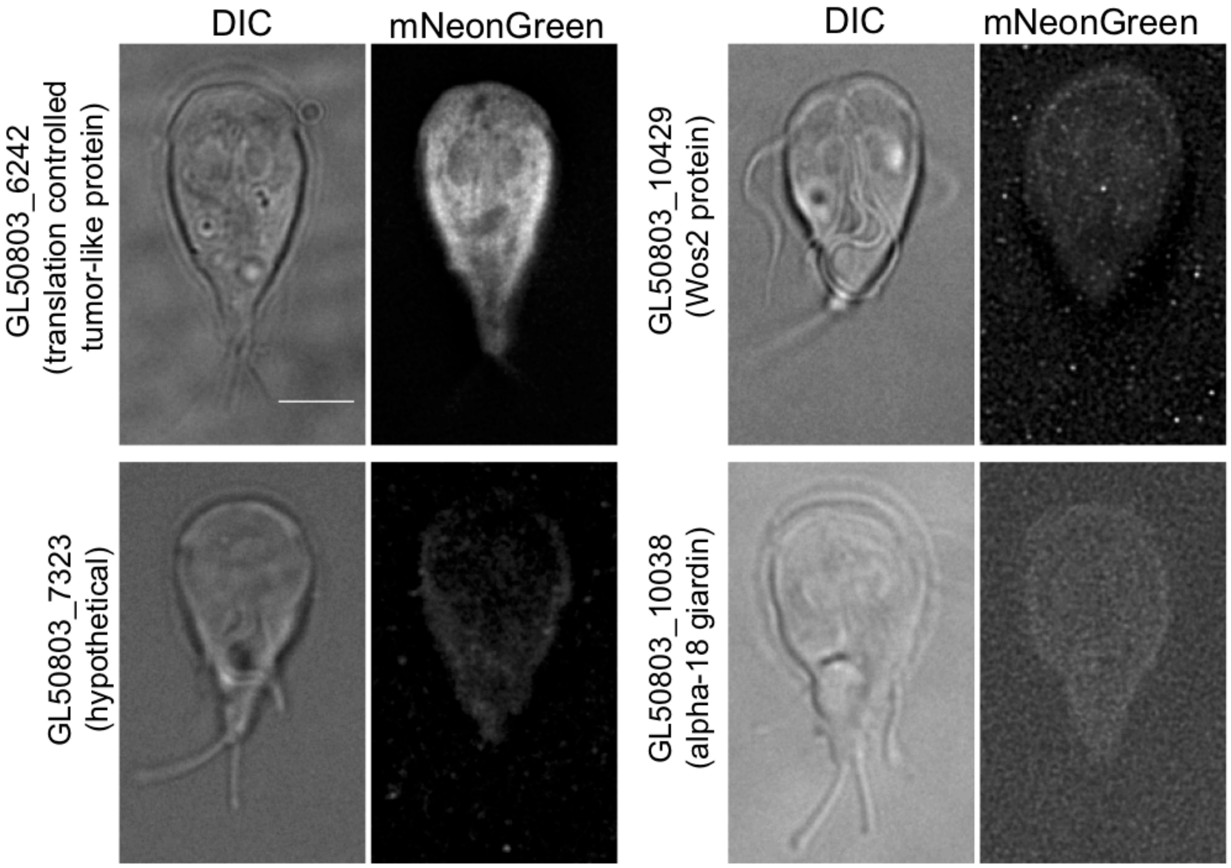

Supplemental Figure 1: Putative *Gl*Actin interactors with non-specific localization or low signal Five proteins had non-specific localization, including faint cytoplasmic localization in GL50803_7323, GL50803_10429, and GL50803_10038. GL50803_6242 and GL50803_16522 were distributed throughout the cytoplasm. Scale bar, 5 µm.

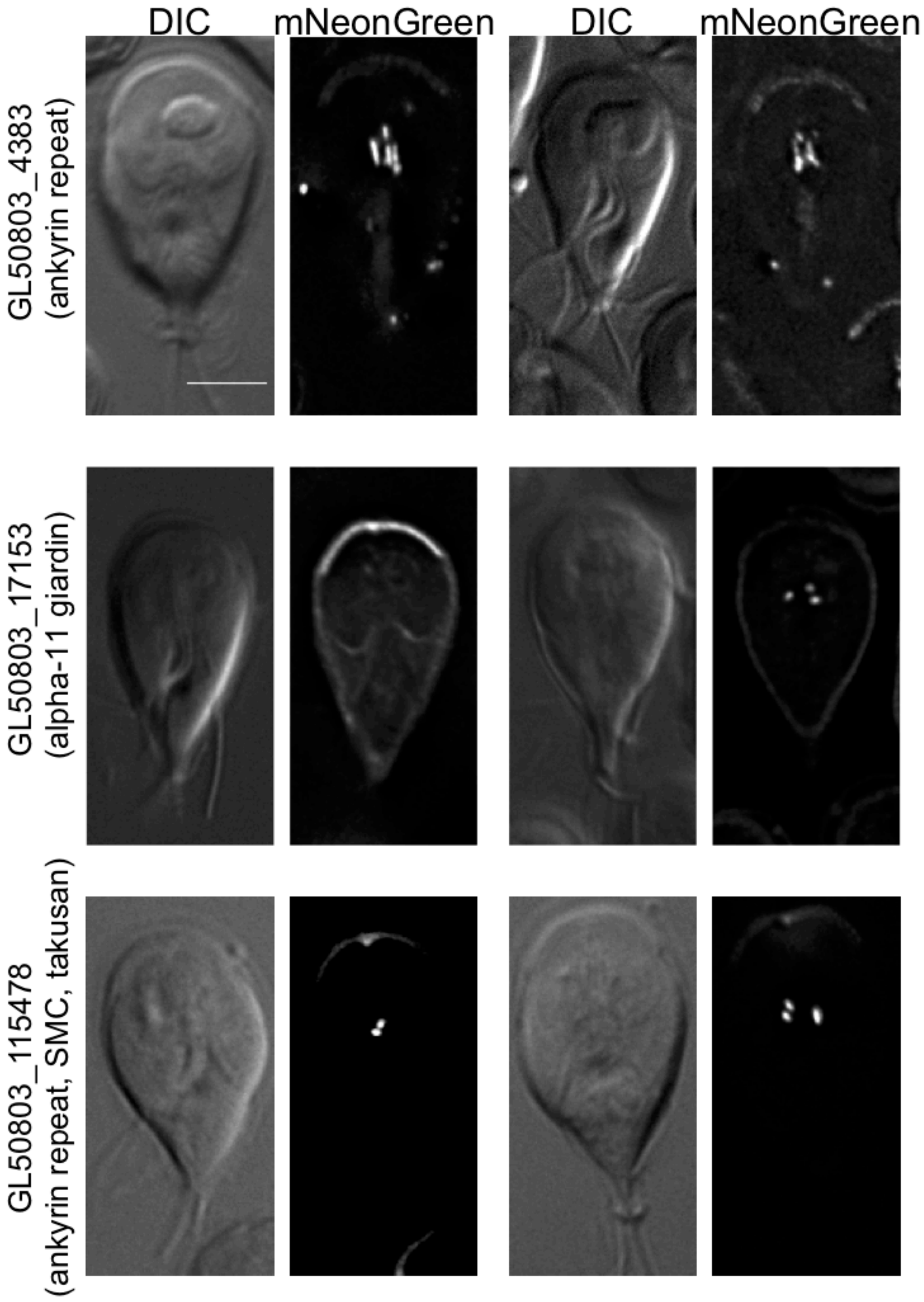

Supplemental Figure 2: Range of mitosome-like localizations in marginal plate proteins

The marginal plate proteins GL50803_17153, GL50803_4383, and GL50803_115478 all localized to axoneme-associated structures which vary in number.
